# Does Breathing through the Mouth Injure the Teeth?

**Published:** 1863-02

**Authors:** Geo. F. Foote


### DOES BREATHING THROUGH THE MOUTH INJURE THE TEETH?

Read before the Cincinnati Dental Society,

BY GEO. F. FOOTE, M. D.

In the economy of man every organ has its specific use, and the perversion of that use is in violation of nature's laws, followed by penalties more or less severe.

The entrance to the mouth is the gateway, and the mouth itself is the high road to the stomach, while the nasal organs are the outer portals to the lungs, both having their specific functions and uses.

Hygienic writers are pointing out to us the evils inflicted upon the general system by a perversion of these uses; that is, by using the mouth as a channel for the ingress and egress of air instead of the nose.

On investigation it will be found that this substitution is very common, and it has occurred to me that *perhaps* we may find in it one of the proximate causes for the early destruction of teeth. I say *perhaps*, for I wish only to call the attention of the profession to this subject, *hoping* that something more tangible may be elucidated by their investigations.

Children and adults often breath through the mouth, while sleeping, as an habitual practice; and many breath through the mouth during their waking hours. By some persons both these are a necessity on account of a real obstruction of the nasal organs, but by most it is only a bad habit, contracted, perhaps, during frequent colds in the head, where, from a slight thickening of the lining membrane, the passage of air through the nose becomes an effort, which, if not overcome, the thickening increases even to an entire obstruction, in consequence of the organ being deprived of its proper stimuli, derived from the passing current.

The teeth, while in situ and possessing vitality, required to be covered with moisture, and kept at the temperature of the body. For the former, nature has been lavish with her secretary organs; and in addition to the moisture found exuding from the mucous surfaces, the saliva is seen pouring in through other channels in sufficient quantities to keep the parts entirely submerged, when the mouth is closed.

In a normal state the mucous secretions are acidulous, while the saliva is alkaline.

Acids destroy both the enamel and ivory of the teeth, while the alkalies do not act upon the enamel, and in its diluted state, found in the saliva, probably not at all upon the ivory possessing vitality. But these should be held in equilibrium.

Any excess of mucous, or the presence of acid, arising from fermentation or other causes, destroys this equilibrium, and is deleterious. The former is evidenced from the soreness of teeth, whose gums are inflamed, and in consequence secrete an abnormal amount of mucous; the latter from the "setting of the teeth on edge" by very acid fruits, and from experiments and observations made upon teeth out of the mouth.

Now then, while breathing through the mouth, particularly when sleeping, the secretion of saliva from the irritating effect of the air is either suspended, or the saliva is evaporated, and the surfaces of the teeth becoming partially dry, gumed up with mucous, and may be particles of food, in a state of decomposition, which the absence of the saliva and the warmth of the mouth promotes, are *perhaps* more or less injured by the destructive effects of these acids.

Again, the currents of air passing over the surfaces of the teeth tend to cool, and impell from them any circulation, causing thereby a suspension of the vital forces that resist chemical unions, while the presence of air promotes the same.

These chemical unions upon the surfaces of the teeth may be exceedingly minute from day to day; yet as nature never repairs those surfaces by restoration, the loss in time becomes serious.

Witness the condition of the teeth during serious illness, particularly of a typhoid diathesis, when the lower jaw, from debility, drops, leaving the mouth open. The mucous surfaces are inflamed; secreting an excess of acidulated mucous; the flow of saliva is partially suspended, and the teeth become sore. The same is observed in ptyalism, either from mercury or other cause, when the abnormal saliva becomes acidulous, which, combining with the acid from the inflamed mucous surfaces, seriously and visibly injure the teeth.

I have now under my care several patients where the incisors have decayed on their labial surfaces, near the gum, all of whom go with their mouths wide open; some until the upper lip has become shortened, exposing these teeth to the atmosphere and thereby depriving them of the neutralizing effect of the saliva.

I have other facts in coroboration of this view which I hope to present in another communication at some future time.

